# Smartphone-Based Indoor Localization with Bluetooth Low Energy Beacons

**DOI:** 10.3390/s16050596

**Published:** 2016-04-26

**Authors:** Yuan Zhuang, Jun Yang, You Li, Longning Qi, Naser El-Sheimy

**Affiliations:** 1 National ASIC System Engineering Research Center, Southeast University, 2 Sipailou, Nanjing 210096, China; zhy.0908@gmail.com (Y.Z.); longn_qi@seu.edu.cn (L.Q.); 2 Department of Geomatics Engineering, The University of Calgary, 2500 University Drive, NW, Calgary, AB T2N 1N4, Canada; liyou331@gmail.com (Y.L.); elsheimy@ucalgary.ca (N.E.-S.); 3 GNSS Research Center, Wuhan University, 129 Luoyu Road, Wuhan 430079, China

**Keywords:** indoor localization, polynomial regression model, fingerprinting, extended Kalman filtering, outlier detection, BLE beacons

## Abstract

Indoor wireless localization using Bluetooth Low Energy (BLE) beacons has attracted considerable attention after the release of the BLE protocol. In this paper, we propose an algorithm that uses the combination of channel-separate polynomial regression model (PRM), channel-separate fingerprinting (FP), outlier detection and extended Kalman filtering (EKF) for smartphone-based indoor localization with BLE beacons. The proposed algorithm uses FP and PRM to estimate the target’s location and the distances between the target and BLE beacons respectively. We compare the performance of distance estimation that uses separate PRM for three advertisement channels (*i.e.*, the separate strategy) with that use an aggregate PRM generated through the combination of information from all channels (*i.e.*, the aggregate strategy). The performance of FP-based location estimation results of the separate strategy and the aggregate strategy are also compared. It was found that the separate strategy can provide higher accuracy; thus, it is preferred to adopt PRM and FP for each BLE advertisement channel separately. Furthermore, to enhance the robustness of the algorithm, a two-level outlier detection mechanism is designed. Distance and location estimates obtained from PRM and FP are passed to the first outlier detection to generate improved distance estimates for the EKF. After the EKF process, the second outlier detection algorithm based on statistical testing is further performed to remove the outliers. The proposed algorithm was evaluated by various field experiments. Results show that the proposed algorithm achieved the accuracy of <2.56 m at 90% of the time with dense deployment of BLE beacons (1 beacon per 9 m), which performs 35.82% better than <3.99 m from the Propagation Model (PM) + EKF algorithm and 15.77% more accurate than <3.04 m from the FP + EKF algorithm. With sparse deployment (1 beacon per 18 m), the proposed algorithm achieves the accuracies of <3.88 m at 90% of the time, which performs 49.58% more accurate than <8.00 m from the PM + EKF algorithm and 21.41% better than <4.94 m from the FP + EKF algorithm. Therefore, the proposed algorithm is especially useful to improve the localization accuracy in environments with sparse beacon deployment.

## 1. Introduction

Currently, indoor localization has become a significant research topic as it is the fundamental of numerous Internet of Things (IoT) applications (e.g., human tracking and precision advertisement) [1]. Several technologies can be used for indoor localization, such as wireless localization [1,2,3,4], sensor-based relative navigation (*i.e.*, dead-reckoning) [5,6,7,8,9,10,11,12], and image-based navigation [13,14,15,16,17]. Wireless localization has been widely used among these technologies. Especially, WiFi localization is the most common consumer wireless localization technology [1,18,19]. Another important candidate for wireless localization on consumer smart devices is Bluetooth. The traditional Bluetooth has a significantly long scan time (~10 s), which limits its value for localization. However, the new Bluetooth protocol (*i.e.*, Bluetooth Low Energy, BLE), supported by most current smart devices, has overcome the limitations of long scan time. Moreover, the BLE beacons have the following advantages: small size, light weight, low cost, power saving and are widely supported by smart devices. Therefore, BLE has the potential to become a dominant wireless localization technology.

In the BLE protocol definition, 40 channels, each 2 MHz wide, around the 2.4 GHz radio band are used to transmit messages. The duration for transmitting messages is extremely short to save battery power. Among these 40 channels, there are three channels (*i.e.*, 37, 38, and 39) for broadcasting advertisement messages. The received signal strengths (RSS) from these three channels can be used for estimating the target’s location. The BLE advertising rate can be set up to 50 Hz. The transmission power for BLE beacons are also set from 0 dBm to −40 dBm. To reduce power consumption, BLE advertising rate and transmission power are usually set to less than 10 Hz and −16 dBm, respectively. Comparing with WiFi localization, BLE localization has the following advantages:

- BLE RSS signals can have a higher sample rate than WiFi RSS signals (0.25 Hz~2 Hz)
- BLE consumes less power than WiFi
- BLE RSS signals can be obtained from most smart devices, while WiFi RSS signals cannot be provided by Apple portable devices and
- BLE beacons are usually battery powered, which are more flexible and easier deployed than WiFi.

Therefore in this paper, we proposes an algorithm for smartphone-based indoor localization using RSS signals from BLE beacons.

Currently, there are mainly three RSS-based BLE localization techniques: proximity, trilateration (or, range-based) and fingerprinting (FP) [20]. The proximity algorithms pre-set an event-triggering threshold for a coverage area. If the RSS values are stronger than the threshold, the target is indicated in the area. In the trilateration algorithm, the propagation model (PM) is used to estimate the distances between the target and BLE beacons. Then, the estimation technique (e.g., least squares or Kalman filtering) is used to estimate the target’s location from these distances and the locations of BLE beacons. FP first builds a radio map database where RSSs from available BLE beacons are mapped to absolute positions. Then, the target’s location is determined by using the average or weighted average of the locations of several most matched fingerprints in the database.

To further improve accuracy and robustness, we propose an algorithm for indoor localization with BLE beacons by using the combination of polynomial regression model (PRM), FP, outlier detection and extended Kalman filtering (EKF). Since indoor environments are complicated by reflection, shadowing and multipath, the radio PM may be not accurate for distance estimation in the indoor environment. Therefore, the proposed algorithm uses PRM to estimate the distances between the target and BLE beacons. Meanwhile, FP is also used to determine the target’s location.

**Figure 1:**
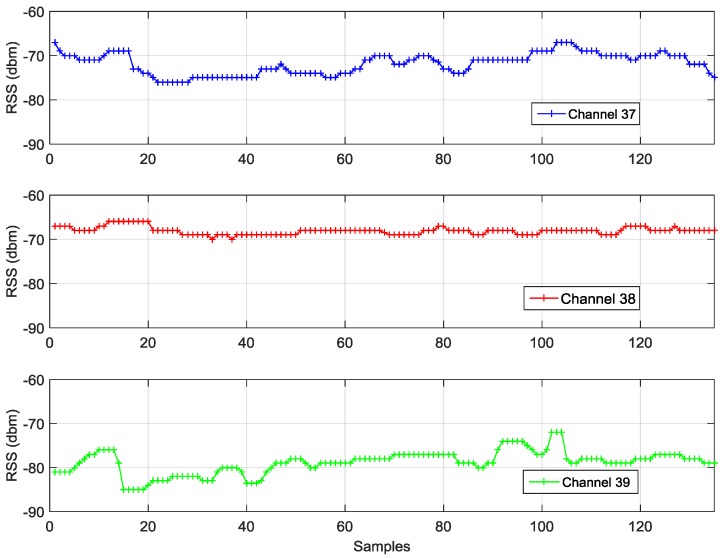
RSS values received from three advertisement channels by keeping the device static.

Most of existing research combines RSS from all channels to obtain an aggregate signal (the aggregate mode). However, we notice that the signals and noises from various advertisement channels may be different, which is shown in Figure 1. In Figure 1, RSS values from three advertisement channels of a BLE beacon were collected by keeping an iPhone 4S static and five meters away. As reported by iOS 8, the mean and standard deviation (STD) of RSS values from various advertisements were different (*i.e.*, Channel 37: Mean −71.9 dBm, STD 2.4 dBm; Channel 38: Mean −68.1 dBm, STD 0.8 dBm; and Channel 39: Mean −78.8 dBm, STD 2.7 dBm). This difference might be caused by the different channel gain and multipath effect. Therefore, one advantage of this research is that it considers that signals and noises in RSS measurements from various channels may be different and thus uses separate PRM and FP for three advertisement channels (*i.e.*, the separate mode), that is, the PRM and FP are used for each advertisement channel separately. Therefore, there are a maximum of three PRM distance estimates for each observed BLE beacon and three FP location estimates. Furthermore, to enhance the robustness of the algorithm, a two-outlier-detection mechanism is designed. These estimates are fed into the first outlier detection to generate improved distance estimates for observed BLE beacons. Next, these improved distance estimates are utilized as measurements for the EKF. Finally, the second outlier detection algorithm is further performed to remove the outliers from the measurements by using the statistical testing method.

The contribution of this paper is listed as follows:

(1) We propose the usage of the separate PRM to improve both the location and distance estimation for each advertisement channel of BLE beacons. Moreover, we originally generate separate radio map database for each BLE advertisement channel for the FP process.
(2) We originally propose an algorithm for BLE-based indoor localization by combing separate PRM, separate FP, EKF and outlier detection.
(3) We propose a two-level outlier detection algorithm to improve the robustness of the system.

The proposed algorithms are validated in detail by field experiments with both sparsely and densely distributed BLE beacons. Part of the outcomes are:

(1) Compared with results that use traditional PM, the distance estimation accuracy is improved by 18.42% using the PRM.
(2) In the case of dense deployment of BLE beacons, the proposed algorithm achieves average 35.82% and 15.77% improvement of the location accuracy in two trajectories, compared with classical PM + EKF and FP + EKF, respectively. The improvement changes to 49.58% and 21.41% in the sparse deployment.

This paper is organized as follows. Section 2 reviews the related work. Section 3 presents the proposed algorithm, Section 4 evaluates the algorithm with various field experiments and Section 5 draws the conclusions.

## 2. Related Work

Current localization algorithms for BLE beacons can be divided into three classes: proximity [21,22,23], range-based [21,24,25,26] and FP [4,21,27,28]. In the first class, the work [21] evaluates the performance of BLE localization by using proximity in a multi-floor building. A triggering threshold optimization method is proposed for proximity based positioning [22]. The research [23] proposes a particle filtering for proximity based indoor positioning. In the second class, a PM is used in a multi-floor building for BLE localization [21]. The research [24] presents a modified PM, called iRingLA, for BLE localization, in which the ring is used instead of circle for trilateration. Another approach called stigmergic is presented for range-based indoor localization [25]. This approach can reduce the effects of multipath, fading, and shadowing for BLE positioning. The work [26] proposes several empirical PMs for BLE based indoor localization in different conditions such as indoor/outdoor and line-of-sight (LOS)/non-line-of-sight (NLOS). In the third class, the research [21] presents a FP for BLE localization in a multi-floor building. The work [27] compares three FP algorithms: k-nearest neighbors (k-NN), neural networks, and support vector machine (SVM). The result shows that k-NN is a good candidate for localization in real-life applications. The research [4] provides a detailed study of the effects of beacon density, transmit power and transmit frequency for BLE FP. A FP solution using Weibull probability distribution is proposed in [28] to improve the reliability and accuracy of the positioning. In addition, the paper [29] presents a comparative analysis of contemporary BLE indoor positioning solutions, taking into account the classification, comparison and various considerations that are required for designing new indoor positioning approaches. All the above research combines RSS from all channels to obtain an aggregate signal, instead of using separate model for each separate BLE advertisement channel.

## 3. Algorithm Description

This section first presents the overview of the whole system. Next, PRM is described for distance estimation, which is followed by FP for location estimation. Then, “Outlier Detection—Level 1” uses the information from PRM and FP to remove the outliers from the measurements and generate improved distance measurements for EKF. Finally, the details of EKF and “Outlier Detection—Level 2” are described to further remove outliers and achieve an enhanced indoor localization solution.

### 3.1. System Overview

**Figure 2:**
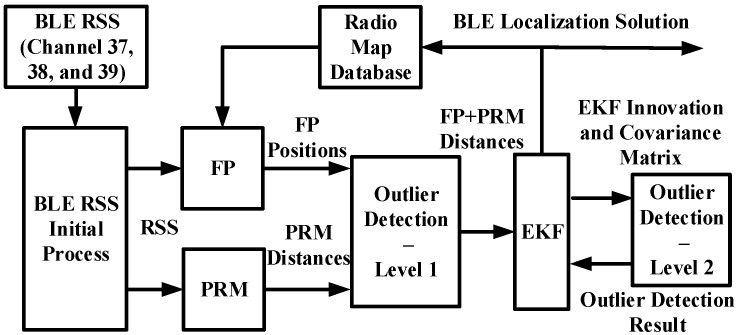
Overview of the smartphone-based indoor localization algorithm with BLE beacons.

The overview of the proposed algorithm is shown in Figure 2, which mainly consists of FP, PRM, EKF, and a two-level outlier detection mechanism. The processed data for the proposed algorithm are RSS values from three advertisement channels of observed BLE beacons. The first process for these RSS values is passing a smoother. Next, we originally use channel-separate models/databases to process RSS values in FP and PRM to generate location and distance estimates. Since BLE RSS values from three advertisement channels are processed separately, there could be three different FP-derived locations and PRM-derived distances. These derived location and distance estimates are then fed into the “Outlier Detection—Level 1” to generate improved FP + PRM distance estimates using statistical methods. The EKF is then used to process these enhanced FP + PRM distance estimates to estimate the target’s location. The second outlier detection based on statistical testing is utilized to further remove the outliers. After the EKF computation with outlier detection, EKF outputs the final BLE localization solution. The final BLE localization solution is also fed to the radio map database to select appropriate fingerprints to improve the FP performance. Overall, the purpose of the proposed algorithm is to achieve a robust and accurate localization solution for the target.

### 3.2. Polynomial Regression Model

In indoor localization using BLE beacons, the radio PM is usually used to model the relationship between measured RSS readings and corresponding distances. The mostly used model is the lognormal shadowing model as follows [30,31]:

(1) $$P\left(d\right)=P\left(d_{0}\right)-10\gamma log_{10}\left(\frac{d}{d_{0}}\right)+X_{\sigma }$$

where γ represents the path-loss exponent, $P\left(d_{0}\right)$ represents the RSS at the reference distance, $d_{0}$, P(d) represents the RSS at the distance between the access point (AP) and the receiver, d, and $X_{\sigma }$ represents a Gaussian random variable, with zero mean, caused by shadow fading [31]. After the model calibration for propagation parameters, the PM model can be used to convert the RSS values to distances between wireless APs and the receiver.

PM is derived from the outdoor environment and works well for LOS scenarios; however, PM may be inaccurate for distance estimation indoors since the indoor environment is complicated by reflection, shadowing and multipath. Actually, indoor wireless signals consist of both LOS and NLOS signals. These signals may have different propagation parameters. Furthermore, it is difficult to differentiate the LOS and NLOS signals in indoor environments. Therefore, the fitted theoretical PM from all training data may not work well for both LOS and NLOS signals and result in large errors in distance estimation.

To overcome this problem, this paper proposes the PRM to model the relationship between RSS and distance for BLE beacons. Different from PM, PRM assumes that the RSS-distance relationship is a nth-degree polynomial and the polynomial coefficients are estimated from the training data in the calibration process. The PRM is given by

(2) $${\hat{d}}_{PRM}=\sum_{i=0}^{n}c_{i}\cdot RSS^{i}$$

where $c_{i}$ are the coefficients of the nth-degree polynomial, RSS is the RSS value, and ${\hat{d}}_{PRM}$ is the estimated distance. The sum of the model fitting error squares is expressed as

(3) $$E\left(c_{0},c_{1},\cdots ,c_{n}\right)=\sum_{j=1}^{M}{\left({\hat{d}}_{PRM,j}-d_{j}\right)}^{2}={\sum_{j=1}^{M}\left(d_{j}-\sum_{i=0}^{n}c_{i}\cdot RSS_{j}^{i}\right)}^{2}$$

where M is the number of the calibration points, $d_{j}$ represents the true distance between the jth calibration point and the BLE beacon, j=1,2,…,M, and $RSS_{j}$ is the RSS at the jth calibration point. From the criterion of least squares estimation, $E\left(c_{0},c_{1},\cdots ,c_{n}\right)$ should be minimized. To achieve the minimum of $E\left(c_{0},c_{1},\cdots ,c_{n}\right)$, we equate its partial derivation to zero with respect to $c_{0},c_{1},\cdots ,c_{n}$, and get

(4) $$\frac{\partial E}{\partial c_{i}}=2\sum_{j=1}^{M}RSS_{j}^{i}\left(d_{j}-\sum_{i=0}^{n}c_{i}\cdot RSS_{j}^{i}\right)=0$$

Equation (4) can be rewritten as follows:

(5) AC=B

where

(6) $$A=\left[\begin{array}{cccc} \sum_{j=1}^{M}RSS_{j}^{0}RSS_{j}^{0} & \sum_{j=1}^{M}RSS_{j}^{0}RSS_{j}^{1} & \cdots & \sum_{j=1}^{M}RSS_{j}^{0}RSS_{j}^{n}\\ \sum_{j=1}^{M}RSS_{j}^{1}RSS_{j}^{0} & \sum_{j=1}^{M}RSS_{j}^{1}RSS_{j}^{1} & \cdots & \sum_{j=1}^{M}RSS_{j}^{1}RSS_{j}^{n}\\ \vdots & \vdots & \cdots & \vdots \\ \sum_{j=1}^{M}RSS_{j}^{n}RSS_{j}^{0} & \sum_{j=1}^{M}RSS_{j}^{n}RSS_{j}^{1} & \cdots & \sum_{j=1}^{M}RSS_{j}^{n}RSS_{j}^{n} \end{array}\right]$$

(7) $$B={\left[\begin{array}{cccc} \sum_{j=1}^{M}RSS_{j}^{0}d_{j} & \sum_{j=1}^{M}RSS_{j}^{1}d_{j} & \cdots & \sum_{j=1}^{M}RSS_{j}^{n}d_{j} \end{array}\right]}^{T}$$

and

(8) $$C={\left[\begin{array}{cccc} c_{0} & c_{1} & \cdots & c_{n} \end{array}\right]}^{T}$$

Finally, the polynomial coefficients can be calculated by

(9) $$C=A^{-1}B$$

After the calibration, PRM can be used to convert RSS values to distances. In this paper, three PRMs are used to estimate distances for three advertisement channels of each BLE beacon. Therefore, there can be three distance estimates for each BLE beacon. When using PRM, the polynomial degree, n, should be carefully determined. If the polynomial degree is small, the distance estimation error will be large. In contrast, if the polynomial degree is large, the computation load is large. Therefore, the polynomial degree is the balance of the distance estimation error and computation load. The field experiments will further discuss the set of the polynomial degree for PRM.

### 3.3. Fingerprinting

The BLE radio map database for FP was constructed by surveying some points’ locations and taking corresponding RSS values. Gaussian Process Regression (GPR) was used to fill the gaps between the surveyed points. We constructed a separate database for each advertisement channel (*i.e.*, the separate mode). Another database was also constructed by dealing all advertisement channels together (*i.e.*, the aggregate mode). In the field experiments, these databases will be used for the evaluation of the FP performance.

**Figure 3:**
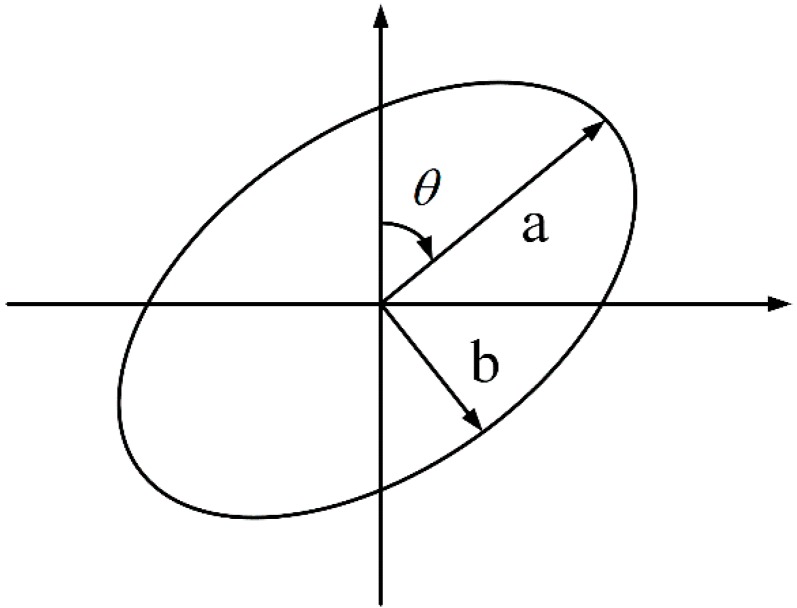
Ellipse-based search space for BLE FP.

On the FP positioning phase, the search space for BLE FP can be limited to an ellipse as determined by the proposed localization solution at the previous epoch, as shown in Figure 3. This localization solution at the previous epoch is used as the center of the ellipse. The major semi-axis, minor semi-axis, and the azimuth of the ellipse are calculated by using the location covariance matrix of the previous epoch from the EKF, that is

(10) $$P=\left[\begin{array}{cc} \sigma_{E}^{2} & \sigma_{EN}\\ \sigma_{NE} & \sigma_{N}^{2} \end{array}\right]$$

where $\sigma_{N}^{2}$ and $\sigma_{E}^{2}$ represent the north and east variances; $\sigma_{NE}$ and $\sigma_{EN}$ are the north/east and east/north covariances. The major semi-axis of the ellipse is

(11) $$a=s\cdot \sqrt{\frac{1}{2}(\sigma_{N}^{2}+\sigma_{E}^{2})+\sqrt{\frac{1}{4}{\left(\sigma_{E}^{2}-\sigma_{N}^{2}\right)}^{2}+\sigma_{NE}^{2}}}$$

The minor semi-axis of the ellipse is

(12) $$b=s\cdot \sqrt{\frac{1}{2}(\sigma_{N}^{2}+\sigma_{E}^{2})-\sqrt{\frac{1}{4}{\left(\sigma_{E}^{2}-\sigma_{N}^{2}\right)}^{2}+\sigma_{NE}^{2}}}$$

And the azimuth of the major semi-axis is

(13) $$\theta =\frac{1}{2}\tan_{4}^{-1}\left(\frac{2\sigma_{NE}^{}}{\sigma_{E}^{2}-\sigma_{N}^{2}}\right)$$

where $\tan_{4}^{-1}(\cdot )$ represents a four-quadrant arctangent operator. s represents the scale factor for the size change of the ellipse, which is set at 5 in this paper. Readers can refer to [32] for the details of the confidence ellipse for measurements.

After the search space is determined, the Euclidean distances are calculated by using current measured fingerprint and the fingerprints in the search space, and the equation is given by

(14) $$ED_{i}=\sqrt{\sum_{j=1}^{N}\frac{{\left(RSS_{m,j}-RSS_{i,j}\right)}^{2}}{N}}$$

where $ED_{i}$ is the Euclidean distance, $RSS_{m}$ is the measured RSS vector, $RSS_{i}$ is the RSS vector of the $i^{th}$ fingerprint in the search space of the radio map database and N is the length of the measured RSS vector. Then, the target’s location is calculated as the weighted average of the locations of the selected fingerprints, and the weight is determined by the inverse of the Euclidean distance. The equation for this calculation is given by

(15) $$r_{FP}=\sum_{i=1}^{K}\frac{\omega_{i}}{\sum_{j=1}^{K}\omega_{j}}r_{i},\;\omega_{i}=\frac{1}{ED_{i}}$$

where $r_{FP}$ is the estimated location of the target, $r_{i}$ is the location of the $i^{th}$ selected fingerprint, $\omega_{i}$ is the weight corresponding to the $i^{th}$ selected fingerprint, $ED_{i}$ is the Euclidean distance between the $i^{th}$ selected fingerprint and the measured fingerprint and K is the total number of selected fingerprints. In this paper, FP is used for each advertisement channel, and can achieve three location estimates for each BLE beacon.

### 3.4. Outlier Detection—Level 1

As per the previous discussion, three PRM-derived distances for each observed BLE beacon and three FP-derived locations can be imported to “Outlier Detection—Level 1” to generate an enhanced FP + PRM distance estimate for each observed BLE beacon. In this paper, three FP-derived locations are represented by $r_{FP37}$, $r_{FP38}$, and $r_{FP39}$. And, three PRM-derived distances are represented by $d_{PM37,i}$, $d_{PM38,i}$, and $d_{PM39,i}$ for the $i^{th}$ BLE beacon. In the first step of “Outlier Detection—Level 1”, FP-derived locations in Equation (15) are used to calculate the FP-derived distances for each observed BLE beacon through the following equation:

(16) $$\begin{array}{l} {\hat{d}}_{FP37,i}=norm\left(r_{FP37}-r_{BLE,i}\right)\\ {\hat{d}}_{FP38,i}=norm\left(r_{FP38}-r_{BLE,i}\right)\\ {\hat{d}}_{FP39,i}=norm\left(r_{FP39}-r_{BLE,i}\right) \end{array}$$

where $r_{BLE,i}$ is the coordinate of the $i^{th}$ BLE beacon. $d_{FP37,i}$, $d_{FP38,i}$, and $d_{FP39,i}$ are three FP-derived distances for the $i^{th}$ BLE beacon. norm is the Euclidean norm (2-norm), and is given by

(17) $$2-norm:{∥x∥}_{2}=\sqrt{\sum_{i=1}^{Nx}{\left|x_{i}\right|}^{2}}$$

where Nx is the length of the vector x, and $x_{i}$ is the term of the vector x.

The statistical method is used to remove the outliers in these six distance estimates: three PRM-derived distance estimates and three FP-derived distances. The confidence interval for these six distance estimates is given by

(18) $${\hat{d}}_{i}\in \left[\begin{array}{cc} \mu_{{\hat{d}}_{i}}-\sigma_{{\hat{d}}_{i}} & \mu_{{\hat{d}}_{i}}+\sigma_{{\hat{d}}_{i}} \end{array}\right]$$

where ${\hat{d}}_{i}$ are the distance estimates (*i.e.*, FP-derived and PRM-derived distances) for the $i^{th}$ BLE beacon. $\mu_{{\hat{d}}_{i}}$ and $\sigma_{{\hat{d}}_{i}}$ are the mean and STD of ${\hat{d}}_{i}$. If any distance estimate is out of the confidence interval, it is moved out as an outlier. Finally, the enhanced FP+PRM distance estimate, ${\hat{d}}_{i,enhanced}$, for the $i^{th}$ BLE beacon is given by

(19) $${\hat{d}}_{i,enhanced}=mean\left({\hat{d}}_{i,trust}\right)$$

where ${\hat{d}}_{i,trust}$ are FP-derived and PRM-derived distance estimates in the confidence interval.

### 3.5. Extended Kalman Filtering

The EKF is used in the proposed algorithm to estimate the target’s current location by fusing current and historical information. In this paper, the state vector of the EKF is given by

(20) $$x={\left[\begin{array}{cccc} r_{e} & r_{n} & v_{e} & v_{n} \end{array}\right]}^{T}$$

where $r_{e}$ and $r_{n}$ are 2D position components (*i.e.*, east and north) in the horizontal plane. $v_{e}$ and $v_{n}$ are their corresponding 2D velocity components. In the proposed algorithm, the EKF system model is the typical kinematic model and is given by

(21) $$x_{k+1|k}=\Phi_{k,k+1}x_{k|k}+\omega_{k}$$

where $x_{k+1|k}$ is the predicted state vector, $x_{k|k}$ is the previous state vector at epoch k and $\Phi_{k,k+1}$ is a 4 × 4 transition matrix:

(22) $$\Phi_{k,k+1}=\left[\begin{array}{cccc} 1 & 0 & \Delta t & 0\\ 0 & 1 & 0 & \Delta t\\ 0 & 0 & 1 & 0\\ 0 & 0 & 0 & 1 \end{array}\right]$$

where Δt is the time difference between two epochs. $\omega_{k}$ is the process noise with the covariance matrix, $Q_{k}=E\left(\omega_{k}\omega_{k}^{T}\right)$, and is given by

(23) $$\omega_{k}={\left[\begin{array}{cccc} 0 & 0 & \varpi_{e,k} & \varpi_{n,k} \end{array}\right]}^{T}$$

where $\varpi_{e,k}$ and $\varpi_{n,k}$ are modelled as white noises for velocity in the east and north directions at epoch k.

The measurement model is given by

(24) $$z_{k}=H_{k}x_{k|k}+\upsilon_{k}$$

where $z_{k}={\left[\begin{array}{cccc} {\hat{d}}_{1,enhanced} & {\hat{d}}_{2,enhanced} & \cdots & {\hat{d}}_{m,enhanced} \end{array}\right]}^{T}$ uses distance estimates from the “Outlier Detection—Level 1” as the measurement vector. $H_{k}$ is the design matrix, and $\upsilon_{k}$ is the measurement noise modelled as a Gaussian white noise, and its covariance matrix is $R_{k}=E\left(\upsilon_{k}\upsilon_{k}^{T}\right)$. In this paper, we define that $r_{e,t}$ and $r_{n,t}$ are 2D position components of the target’s location, which are predicted by the EKF; $r_{e,i}$ and $r_{n,i}$, i=1,2,…,m, are 2D position components of the BLE beacons’ locations. The design matrix is given by

(25) $$H_{k}=\left[\begin{array}{cccc} -\frac{r_{e,1}-r_{e,t}}{\sqrt{{\left(r_{e,1}-r_{e,t}\right)}^{2}+{\left(r_{n,1}-r_{n,t}\right)}^{2}}} & -\frac{r_{n,1}-r_{n,t}}{\sqrt{{\left(r_{e,1}-r_{e,t}\right)}^{2}+{\left(r_{n,1}-r_{n,t}\right)}^{2}}} & 0 & 0\\ -\frac{r_{e,2}-r_{e,t}}{\sqrt{{\left(r_{e,2}-r_{e,t}\right)}^{2}+{\left(r_{n,2}-r_{n,t}\right)}^{2}}} & -\frac{r_{n,2}-r_{n,t}}{\sqrt{{\left(r_{e,2}-r_{e,t}\right)}^{2}+{\left(r_{n,2}-r_{n,t}\right)}^{2}}} & 0 & 0\\ & \vdots & & \\ -\frac{r_{e,m}-r_{e,t}}{\sqrt{{\left(r_{e,m}-r_{e,t}\right)}^{2}+{\left(r_{n,m}-r_{n,t}\right)}^{2}}} & -\frac{r_{n,m}-r_{n,t}}{\sqrt{{\left(r_{e,m}-r_{e,t}\right)}^{2}+{\left(r_{n,m}-r_{n,t}\right)}^{2}}} & 0 & 0 \end{array}\right]$$

There are two phases in the EKF process: prediction and update. In the prediction process, the state vector and covariance matrix are predicted from the system model:

(26) $$\{\begin{array}{c} x_{k+1|k}=\Phi_{k,k+1}x_{k|k}\\ P_{k+1|k}=\Phi_{k,k+1}P_{k|k}\Phi_{k,k+1}^{T}+Q_{k} \end{array}$$

In the update process, the state vector and covariance matrix are updated from the measurement model:

(27) $$\{\begin{array}{c} K_{k}=P_{k|k-1}H_{k}^{T}{\left(H_{k}P_{k|k-1}H_{k}^{T}+R_{k}\right)}^{-1}\\ x_{k|k}=x_{k|k-1}+K_{k}\left(z_{k}-H_{k}x_{k|k-1}\right)\\ P_{k|k}=\left(I-K_{k}H_{k}\right)P_{k|k-1}{\left(I-K_{k}H_{k}\right)}^{T}+R_{k} \end{array}$$

where $K_{k}$ is the Kalman gain.

### 3.6. Outlier Detection—Level 2

In this section, statistical testing on the innovations of the EKF is presented as the second level of outlier detection. When using EKF, we assume the following two conditions: (1) the measurement noise is zero-mean, white and Gaussian distributed; and (2) the process noise is zero-mean, white, and Gaussian distributed. Based on these assumptions, the innovation sequence will be zero-mean, white and Gaussian distributed. The equation for the innovation sequence can be given as

(28) $$\tau_{k}=z_{k}-{\hat{z}}_{k|k-1}$$

where $\tau_{k}$ is the innovation, $z_{k}$ is the observed measurement, and ${\hat{z}}_{k|k-1}=H_{k}x_{k|k-1}$ is the predicted measurement. The innovations have the following covariance matrix [33]:

(29) $$C_{\tau_{k}}=H_{k}P_{k|k-1}H_{k}^{T}+R_{k}$$

where $C_{\tau_{k}}$ is the covariance matrix of the innovation, $H_{k}$ is the design matrix, $P_{k|k-1}$ is the state covariance matrix and $R_{k}$ is the measurement covariance matrix. Given the assumptions stated above, the innovation sequence is distributed as

(30) $$\tau_{k}\sim N\left(0,C_{\tau_{k}}\right)$$

where $N\left(\mu ,C_{\sigma }\right)$ represents the normal distribution with mean of μ and covariance of $C_{\sigma }$. The confidence intervals for the individual measurements are then calculated [33]. If these are violated, the measurement is considered as an outlier, and removed from the EKF.

## 4. Field Experiments

### 4.1. Experimental Setup

**Figure 4:**
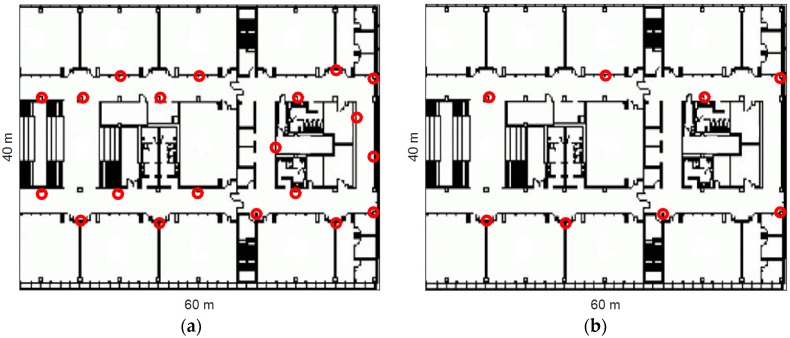
Experimental area (red circles = BLE beacons): (**a**) dense deployment (1 beacon per 9 m); (**b**) sparse deployment (1 beacon per 18 m).

To evaluate the performance of the proposed algorithm, we conducted experiments in an office environment (60 m × 40 m). These experiments were conducted with two different deployments of BLE beacons as shown in Figure 4: (a) dense deployment (total: 20 beacons; average: 1 beacon per 9 m) and (b) sparse deployment (total: 8 beacons; average: 1 beacon per 18 m). All these beacons were installed on the wall at a height of approximately 1.5 m. To balance the power consumption and accuracy, each beacon was set to 10 Hz sample rate with −16 dBm transmit power [4]. The locations of BLE beacons were measured by using a commercial laser rangefinder and the floor plan of the building. Since the floor plan was verified to be very accurate (which had been used for engineering construction), the location errors of BLE beacons were less than 1 m. Two different iPhone 4S with iOS 8 were used by different testers in the experiments. The BLE beacons used in the experiments are based on Texas Instruments (TI) BLE chip CC2540. This chip broadcasts channel information and RSS value together for each advertisement channel, which can be received by an iOS device when using iOS 7 or above.

Many trajectories were tested in the experiments and two of them were selected for performance demonstration in this paper. Several reference points with surveyed locations were used to generate the ground truth. A stopwatch was used to record the time when the testers passed these reference points and thereafter, the locations of the ground truth between these reference points were generated through interpolation. The interpolation method used the stopwatch information and assumed the person walked with a constant speed between two reference points, which as per the instructions to testers when collecting the data.

### 4.2. Performance of Polynomial Regression Model for Distance Estimation

**Figure 5:**
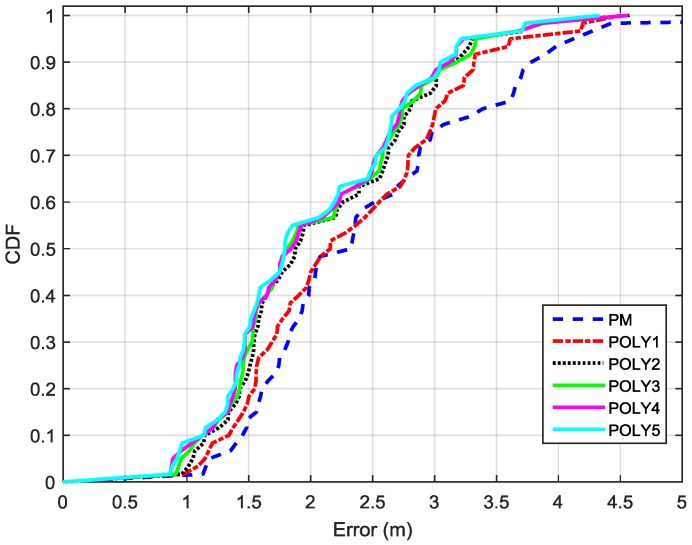
CDFs of 60 average distance estimation errors corresponding to 60 channel models for PM, POLY1, POLY2, POLY3, POLY4, and POLY5.

The performance of PRM-based distance estimation is discussed in this section. First, we need to determine the polynomial degree, n, for the PRM. As per the previous discussion, the selection of the polynomial degree is a trade-off between distance accuracy and computation load. We collected RSS values from BLE beacons at several points with known locations in the dense deployment shown in Figure 4. This data was used to estimate the PM and PRM with different polynomial degrees for each advertisement channel of the BLE beacons. In the estimation, the selected range for polynomial degree is set to n=1,2,…,5, and the corresponding PRM models are called POLY1, POLY2, POLY3, POLY4, and POLY5. In the dense deployment, there are total 60 advertisement channels (*i.e.*, 3 channels/beacon × 20 beacons = 60 channels). Therefore, there are 60 channel models in total for each method (*i.e.*, PM, POLY1, POLY2, POLY3, POLY4 and POLY5). Each channel model has an average distance estimation error, therefore there are a total of 60 average distance estimation errors for each method (*i.e.*, PM, POLY1, POLY2, POLY3, POLY4, and POLY5). The cumulative distribution function (CDF) of these 60 average distance estimation errors for each method, are plotted in Figure 5. As shown in Figure 5, POLY2, POLY3, POLY4 and POLY5 have similar performance and are better than POLY1 and PM. On the other hand, POLY2 has the smallest computation load among these four methods. Therefore, POLY2 is selected for the PRM and PRM represents POLY2 for the rest of the paper. From Figure 5, we can find that the 90% distance estimation error using PRM is 3.1 m, which is reduced by 18.42% over the PM (*i.e.*, 3.8 m).

For the demonstration purpose, one BLE channel is used as an example to compare the PRM and PM. The PRM and PM functions are given by

(31) $$d=0.01582\times RSS^{2}-0.03178\times RSS+0.06341$$

and

(32) $$RSS=-79.5891-10\times 1.2954\times log_{10}\left(d\right)$$

**Figure 6:**
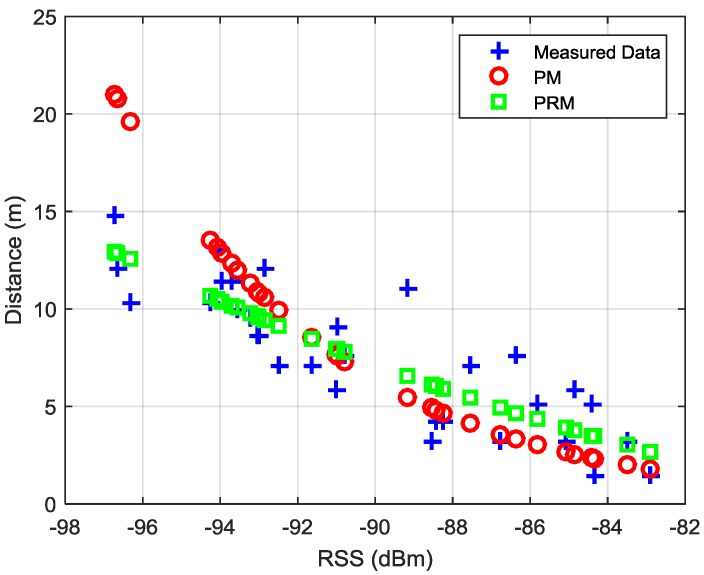
Measured data and distance estimation results.

**Figure 7:**
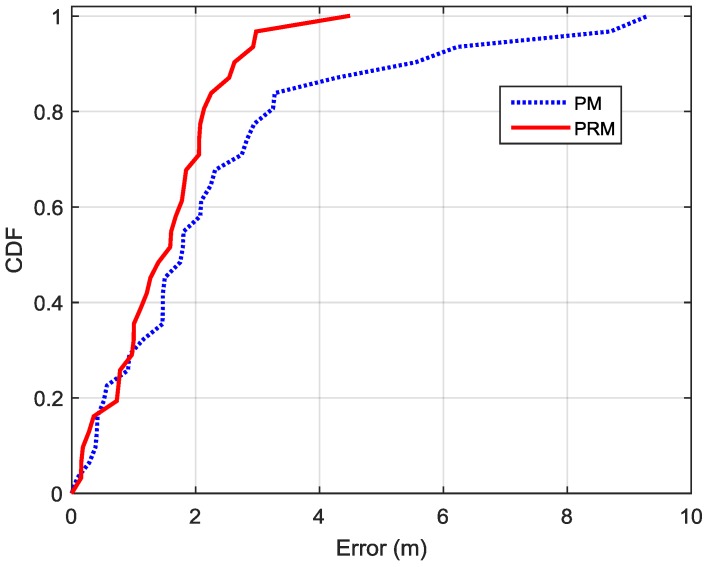
CDFs of distance estimation errors for the selected channel by using PM and PRM.

Figure 6 shows the measured data and distance estimation results using PM and PRM, in which PRM works better than PM in indoor environments. It seems that the relationship between BLE RSS and distance does not obey the log path-loss model in complex indoor environments due to the effect of fading, reflection, multipath, *etc*. Figure 7 depicts the CDF functions of the distance estimation errors in the selected channel using PM and PRM. The 90% error of distance estimation using PRM is 2.6 m, which is only 47.24% of that uses PM (5.5 m).

**Figure 8:**
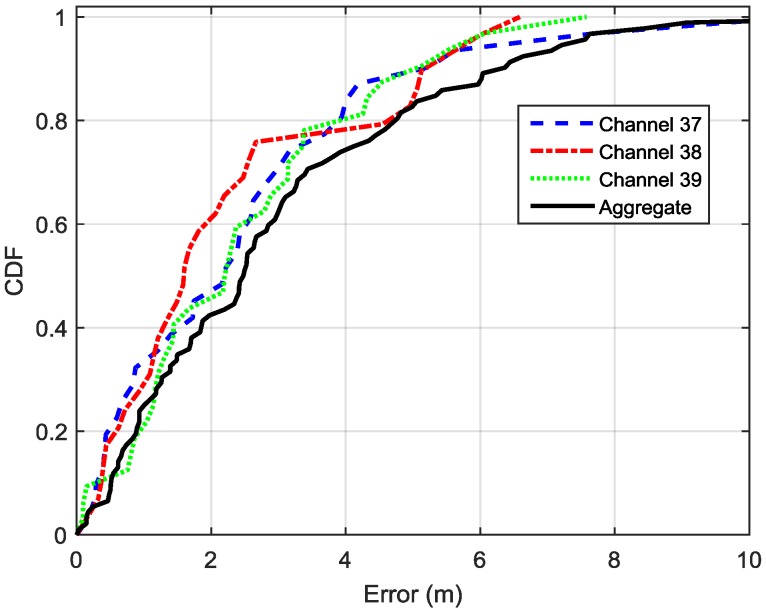
CDFs of distance estimation errors for the selected BLE beacon by using separate PRM and aggregate PRM.

Next, we compare the performance of the distance estimation using the separate PRM for each advertisement channel with that uses the aggregate PRM for all advertisement channels. For the illustration purpose, one BLE beacon is used as an example for this comparison. Figure 8 depicts CDF functions of the distance estimation errors for the selected BLE beacon using separate PRM and aggregate PRM. The 90% distance estimation errors of three separate PRMs are 5.1 m, 5.2 m and 5.0 m; which are decreased by 19.05%, 17.46% and 20.63% over the aggregate PRM (6.3 m) respectively. Therefore, our tests indicate that separate PRM can provide more accurate distance estimation than the aggregate PRM. Thus, separate PRM is adopted in this paper.

### 4.3. Performance of of Fingerprinting for Location Estimation

**Figure 9:**
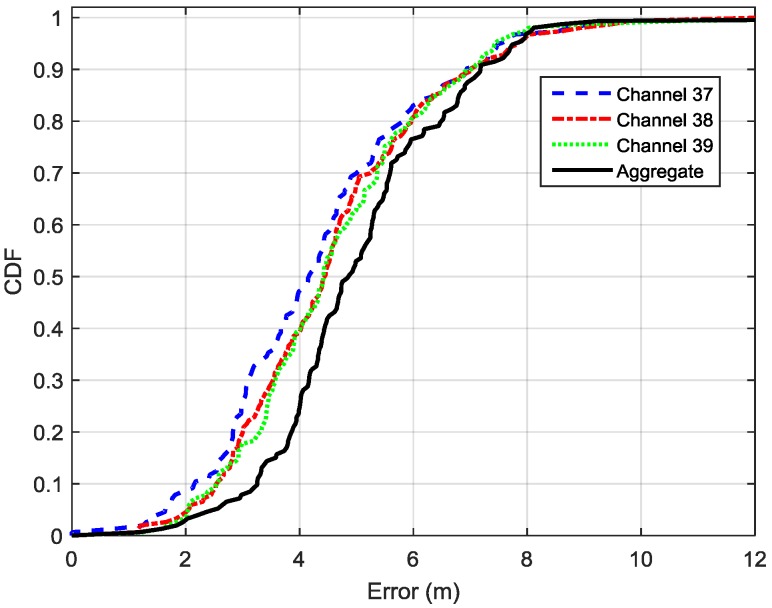
CDFs of location estimation errors by using separate FP databases and aggregate FP database.

This section mainly compares the FP-based location estimation using separate databases for each advertisement channel with that uses the aggregate database. In the comparison, RSS values were collected at 150 points in the experimental area. These RSS values were used for FP calculation by using two types of FP databases: the separate databases and aggregate database. Furthermore, the parameter “k” of k-NN is temporarily set to 1 here to avoid the effect of k-NN for the comparison of these two kinds of databases. To achieve a better localization performance, k is set to 3 in other parts of this paper. Figure 9 depicts CDF functions of the location estimation errors using separate FP databases and aggregate FP database. The FPs using separate databases have better performance than that uses aggregate database. The 90% errors of FP using separate databases are 6.9 m, 7.0 m, and 7.0 m; which are reduced by 4.17%, 2.78%, and 2.78% over that uses the aggregate database (7.2 m), respectively. These tests indicate that the separate strategy can provide more accurate FP result than the aggregate strategy. Thus, FP using separate databases are used in this paper.

### 4.4. Performance Evaluation for the Proposed Algorithm

**Figure 10:**
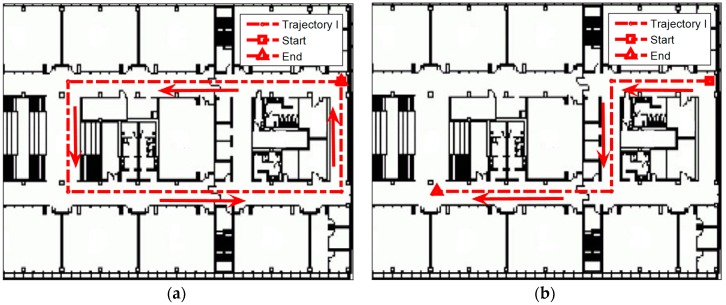
Two trajectories for performance evaluation: (**a**) Trajectory I; and (**b**) Trajectory II.

**Figure 11:**
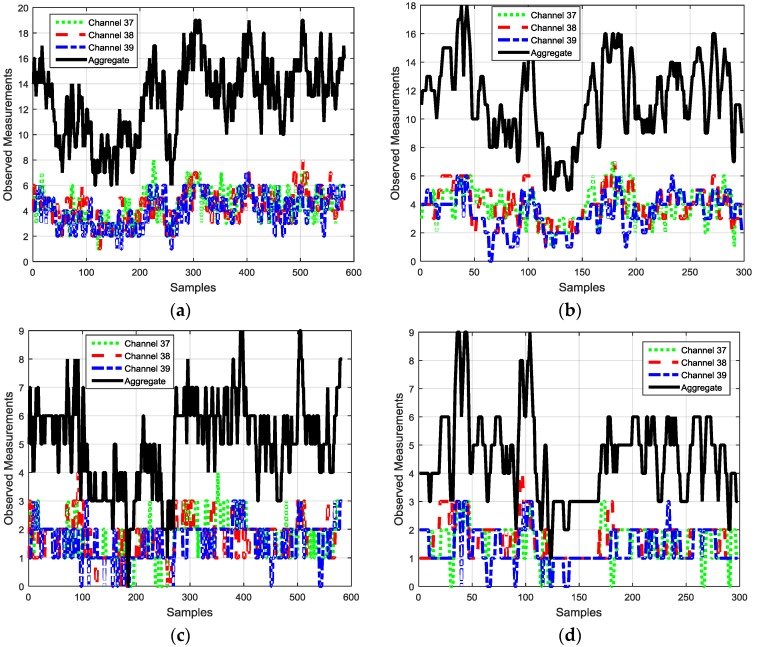
Numbers of observed advertisement channels of BLE beacons in two trajectories with dense and sparse deployment of BLE beacons: (**a**) Trajectory I with 20 deployed BLE beacons; (**b**) Trajectory II with 20 deployed BLE beacons; (**c**) Trajectory I with 8 deployed BLE beacons; and (**d**) Trajectory II with 8 deployed BLE beacons.

The performance of the proposed algorithm was evaluated in indoor environments with densely and sparsely distributed BLE beacons shown in Figure 4. The proposed algorithm was also compared with classical PM + EKF and FP + EKF algorithms. Figure 10 shows the two trajectories which were used for performance evaluation. Figure 11 depicts the numbers of observed advertisement channels from BLE beacons in these two trajectories with dense and sparse deployment of BLE beacons. In Figure 11, there are 5~19 observed advertisement channels in two trajectories with dense deployment while the number changes to 0~9 with sparse deployment. The different number of measurements leads to various localization performance.

**Figure 12:**
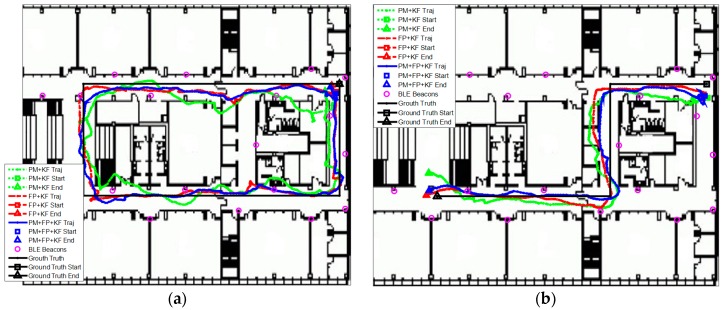
Estimated trajectories with dense deployment of BLE beacons using the proposed algorithm, PM + EKF, and FP + EKF: (**a**) Trajectory I; and (**b**) Trajectory II.

**Figure 13:**
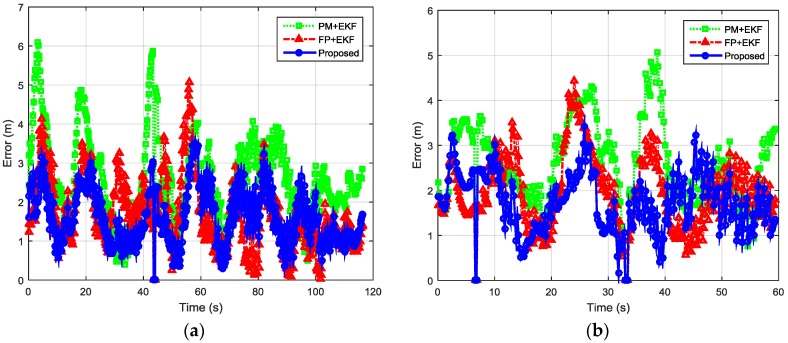
Localization errors of two indoor trajectories with densely distributed BLE beacons using the proposed method, PM + EKF, and FP + EKF: (**a**) Trajectory I; and (**b**) Trajectory II.

**Figure 14:**
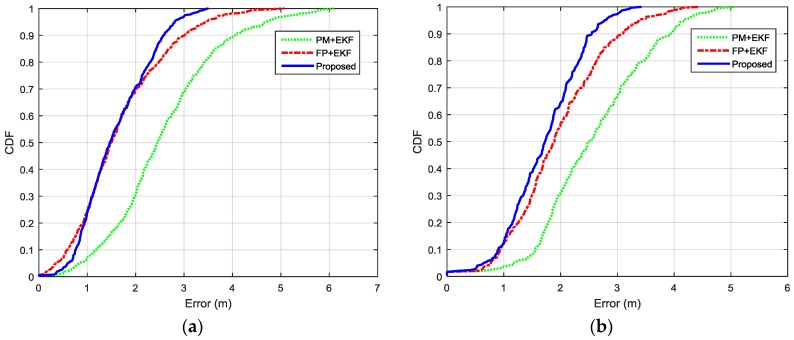
CDFs of localization errors of the two indoor trajectories with densely distributed BLE beacons using the proposed method, PM+EKF, and FP + EKF: (**a**) Trajectory I; and (**b**) Trajectory II.

The localization performance with dense deployment of BLE beacons is discussed first. Figure 12 shows the estimated trajectories using the proposed algorithm, PM + EKF and FP + EKF. The proposed algorithm performs slightly better than FP + EKF and PM + EKF. Figure 13 shows the localization errors of the two trajectories using the proposed method, PM + EKF and FP + EKF in the environment with densely distributed BLE beacons. As shown in Figure 13, the proposed algorithm has less localization errors than PM + EKF and FP + EKF at most of the time. Table 1 summarizes the localization errors of the two trajectories using the proposed algorithm, PM + EKF and FP + EKF. Figure 14 shows the CDF functions of localization errors of the two trajectories using these three approaches. As shown in Figure 14 and Table 1, the 90% localization error of the proposed algorithm is 2.57 m in trajectory I, which is reduced by 36.70% over PM + EKF (4.06 m) and 14.33% over FP + EKF (3.00 m). The 90% localization error of the proposed algorithm is 2.55 m in trajectory II, which reduces by 34.95% over PM + EKF (3.92 m) and 17.21% over FP + EKF (3.08 m). These results demonstrate that the proposed algorithm achieves around 2.5 m 90% localization error in the two trajectories, which performs better than both the traditional PM + EKF and FP + EKF when there is dense deployment of BLE beacons.

**Figure 15:**
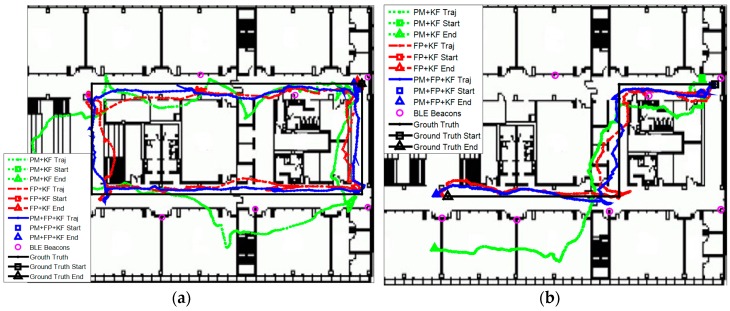
Estimated trajectories with sparse deployment of BLE beacons using the proposed algorithm, PM + EKF and FP + EKF: (**a**) Trajectory I; and (**b**) Trajectory II.

**Figure 16:**
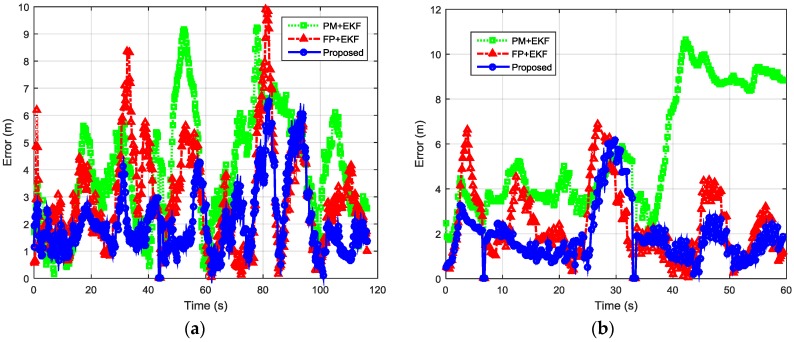
Localization errors of two indoor trajectories with sparsely distributed BLE beacons using the proposed method, PM + EKF and FP + EKF: (**a**) Trajectory I; and (**b**) Trajectory II.

**Figure 17:**
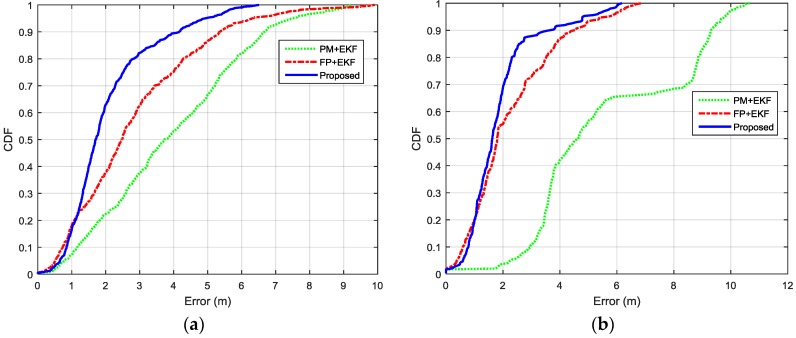
CDFs of localization errors of the two indoor trajectories with sparsely distributed BLE beacons using the proposed method, PM + EKF and FP + EKF: (**a**) Trajectory I; and (**b**) Trajectory II.

Then, we evaluated the localization performance with the sparse deployment of BLE beacons. Figure 15 shows the estimated trajectories with sparsely distributed BLE beacons using the proposed algorithm, PM + EKF, and FP + EKF. The proposed algorithm performs better than FP + EKF and PM + EKF and the improvement is more than the case of dense deployment. Figure 16 shows the localization errors of the two trajectories using the proposed method, PM + EKF, and FP + EKF in the environment with the sparse distribution of BLE beacons. Table 2 summarizes the location errors of the two trajectories in this case. Figure 17 shows the CDF functions of localization errors of the two trajectories using the proposed method, PM + EKF, and FP + EKF. In Figure 17 and Table 2, the 90% localization error of the proposed algorithm is 4.16 m in trajectory I, which is reduced by 37.72% over PM + EKF (6.68 m) and 22.24% over FP + EKF (5.35 m). Figure 17 and Table 2 also show that the 90% localization error of the proposed algorithm is 3.59 m in trajectory II, which is decreased by 61.44% over PM + EKF (9.31 m) and 20.58% over FP + EKF (4.52 m). These results demonstrate that the proposed algorithm achieves around 4.0 m 90% localization error in the two trajectories with the sparse deployment of BLE beacons, which performs much better than the traditional PM + EKF and FP + EKF. The improvement of the proposed method (37.72% and 61.44% for PM + EKF, and 22.24% and 20.58% for FP + EKF) in two trajectories with the sparse deployment is more than that (36.70% and 34.95% for PM + EKF, and 14.33% and 17.21% for FP + EKF) with the dense deployment. This outcome means that the proposed algorithm is especially useful to improve the localization accuracy in the environments with sparse beacon deployment.

Table 3 compares the localization errors of the proposed method in two cases: (a) dense deployment and (b) sparse deployment. It shows that the 90% localization errors with dense deployment are 2.57 m and 2.55 m in two trajectories, which are less than the 4.16 m and 3.59 m of the sparse deployment. The 90% localization errors with sparse deployment are increased by 61.87% and 40.78% over that with dense deployment in the two trajectories, respectively. This result illustrates that the proposed method in the dense deployment performs better than the sparse deployment. However, the dense deployment has more expense. Therefore, it is suggested that the deployment of BLE beacons should comprehensively consider both requirement of the localization accuracy and budget in specific indoor positioning and navigation applications.

## 5. Conclusions

This paper proposed an innovative algorithm based on the integration of channel-separate polynomial regression model (PRM), channel-separate fingerprinting (FP), extended Kalman filtering (EKF), and outlier detection for indoor localization using Bluetooth Low Energy (BLE) beacons. Field experiments showed that the proposed PRM for distance estimation achieved the accuracies of <3.1 m at 90% of the time, which was reduced by 18.42% over the 3.8 m of traditional propagation model (PM). Also, the proposed algorithm provided the accuracies of <2.56 m at 90% of the time (average of two trajectories) with dense deployment of BLE beacons (1 beacon per 9 m), which performed better than <3.99 m of the classical PM + EKF algorithm and <3.04 m of the classical FP + EKF algorithm. With sparse deployment of BLE beacons (1 beacon per 18 m), the proposed algorithm achieved the accuracies of <3.88 m at 90% of the time (average of two trajectories), which performed better than <8.00 m of the classical PM + EKF algorithm and <4.94 m of the classical FP + EKF algorithm. The improvement of the proposed algorithm over the classical PM + EKF and FP + EKF with sparse deployment of BLE beacons was more significant than the case of dense deployment. This proposed algorithm can be easily implemented without extra hardware costs and promote the development of robust smartphone-based indoor localization systems with BLE beacons.

## Figures and Tables

**Table 1:** Summary of localization errors of two trajectories in the case of dense distribution of BLE beacons (m).

Trajectory	Algorithm	50%	90%	Mean	RMS
I	PM + EKF	2.44	4.06	2.57	2.81
	FP + EKF	1.48	3.00	1.67	1.91
	Proposed	1.46	2.57	1.59	1.74
II	PM + EKF	2.49	3.92	2.59	2.76
	FP + EKF	1.89	3.08	1.96	2.12
	Proposed	1.72	2.55	1.72	1.84

**Table 2:** Summary of localization errors of the two trajectories in the case of sparse distribution of BLE beacons (m).

Trajectory	Algorithm	50%	90%	Mean	RMS
I	PM + EKF	3.72	6.68	3.93	4.46
	FP + EKF	2.47	5.35	2.83	3.40
	Proposed	1.70	4.16	2.07	2.44
II	PM + EKF	4.60	9.31	5.59	6.20
	FP + EKF	1.80	4.52	2.27	2.75
	Proposed	1.63	3.59	1.89	2.27

**Table 3:** Summary of localization errors of the two trajectories using the proposed algorithm with both dense and sparse distribution of BLE beacons (m).

Trajectory	Algorithm	50%	90%	Mean	RMS
I	Dense Distribution	1.46	2.57	1.59	1.74
	Sparse Distribution	1.70	4.16	2.07	2.44
II	Desnse Distribution	1.72	2.55	1.72	1.84
	Sparse Distribution	1.63	3.59	1.89	2.27
